# Supplementary material for: Models for the architecture of the human inner kinetochore on centromeric α-satellite CENP-A nucleosome arrays
Source: Nat Commun. 2026 May 12;17:6346. doi: 10.1038/s41467-026-72856-0 (PMC13376190; doi:10.1038/s41467-026-72856-0)
Supplement: Supplementary file 1 — Supplementary Information [file 41467_2026_72856_MOESM1_ESM.pdf]

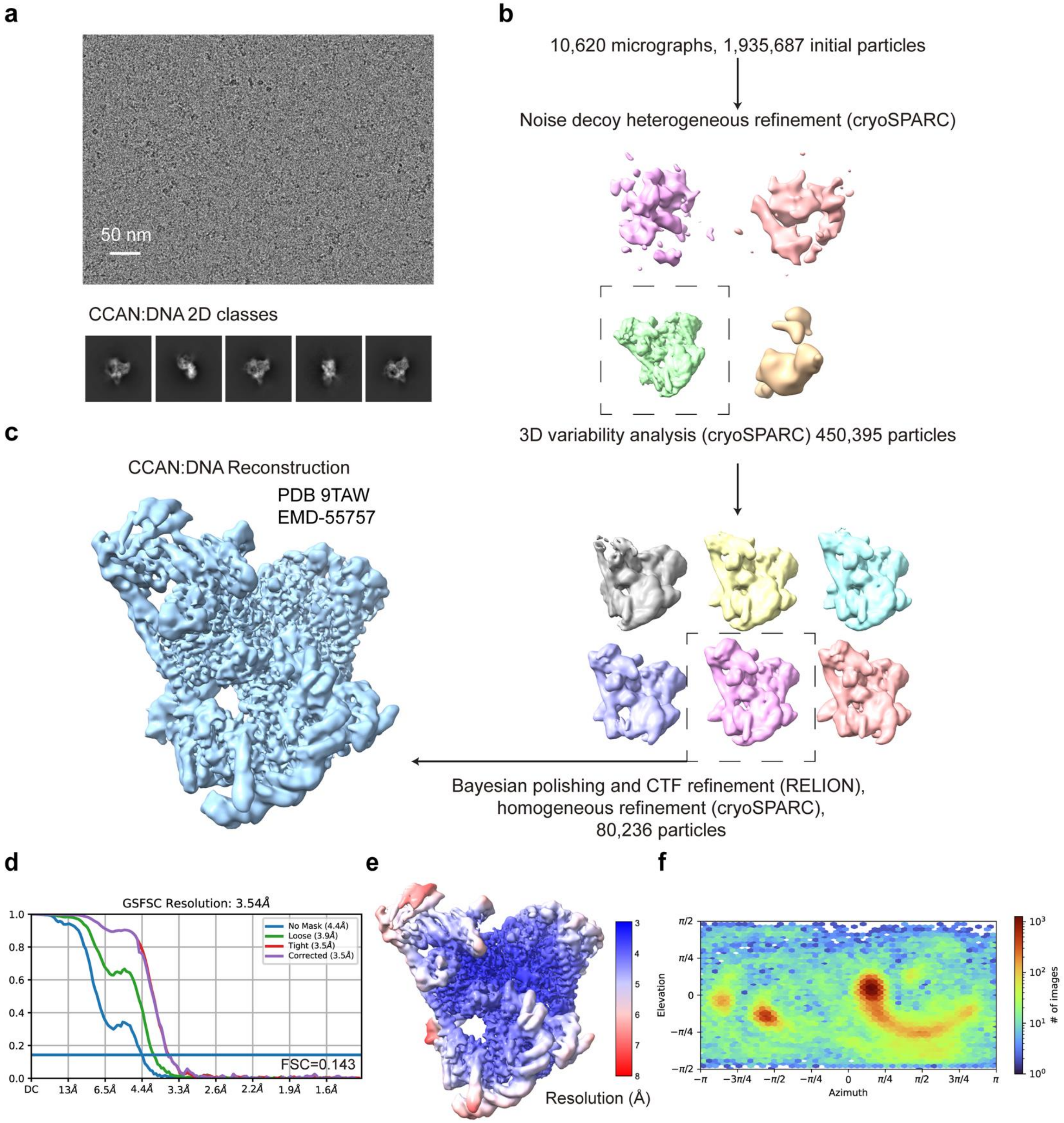

Supplementary Figure 1. Cryo-EM data and processing workflow for the CCAN:DNA complex. **a**, Representative cryo-electron micrograph of 10,628 collected and 2D class averages for CCAN:DNA. **b**, Cryo-EM processing workflow. **c**, 3D reconstruction. A sharpened map is shown in Fig. 1c. **d**, FSC curves. **e**, Cryo-EM reconstruction colour-coded according to local resolution. **f**, Plot of the angular distribution of particles used in the final reconstruction.

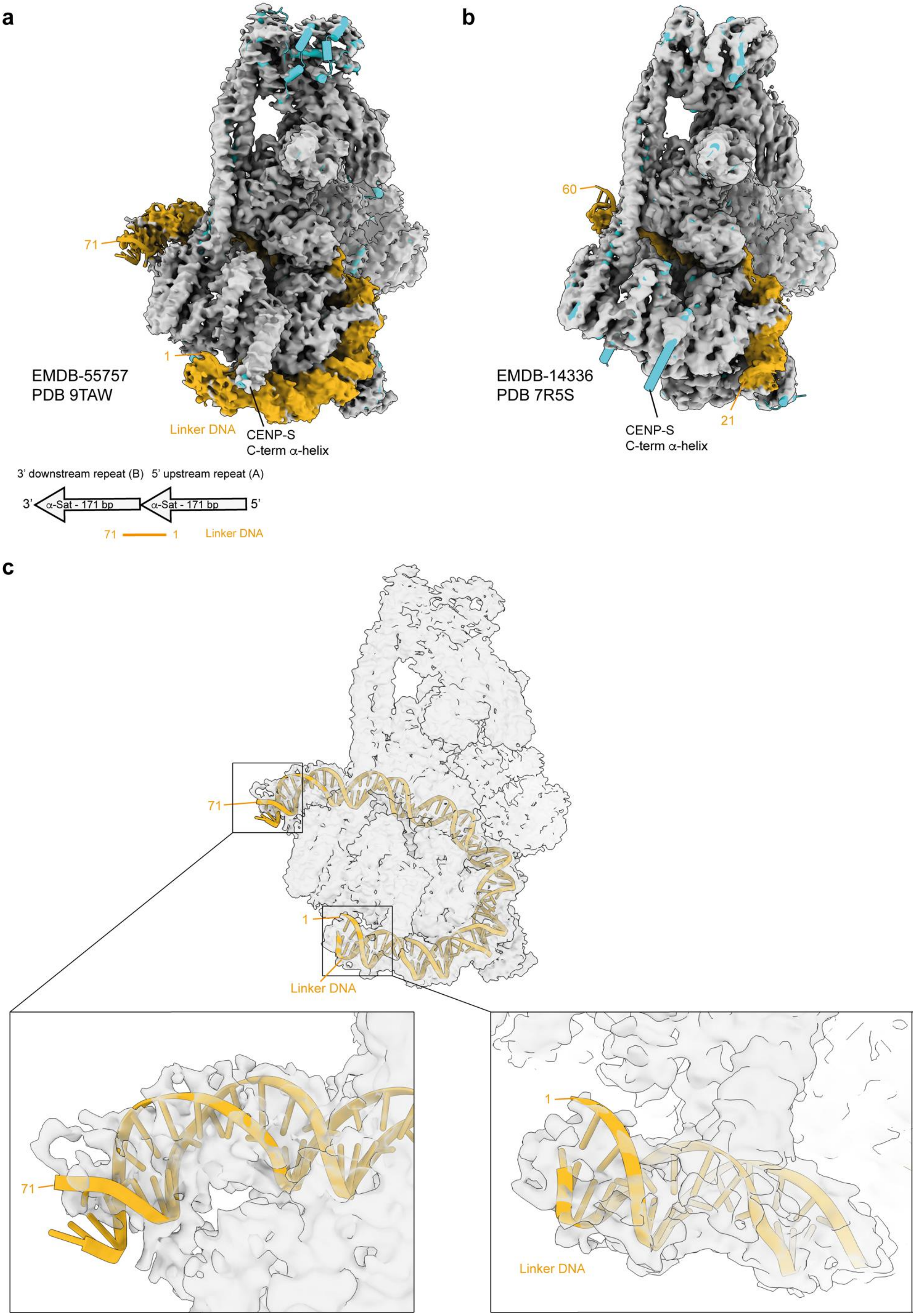

Supplementary Figure 2. Cryo-EM density fits for DNA to the CCAN:DNA complex. a, Cryo-EM map of CCAN:DNA complex with CCAN density in grey and DNA density in orange. CCAN is shown as a cyan-coloured ribbon. 70 bp of DNA are resolved in density. **b**, Cryo-EM map of previous CCAN:DNA complex using 54 bp DNA which has 40 bp ordered [12]. Base pair numbering matches (**a**). **c**, Cryo-EM map of CCAN:DNA complex shown with a transparent surface and DNA shown as ribbons in gold to indicate ordered DNA density fit to coordinates. Below: panels showing the termini of the DNA as indicated in the 'boxed' regions in the overview panel above.

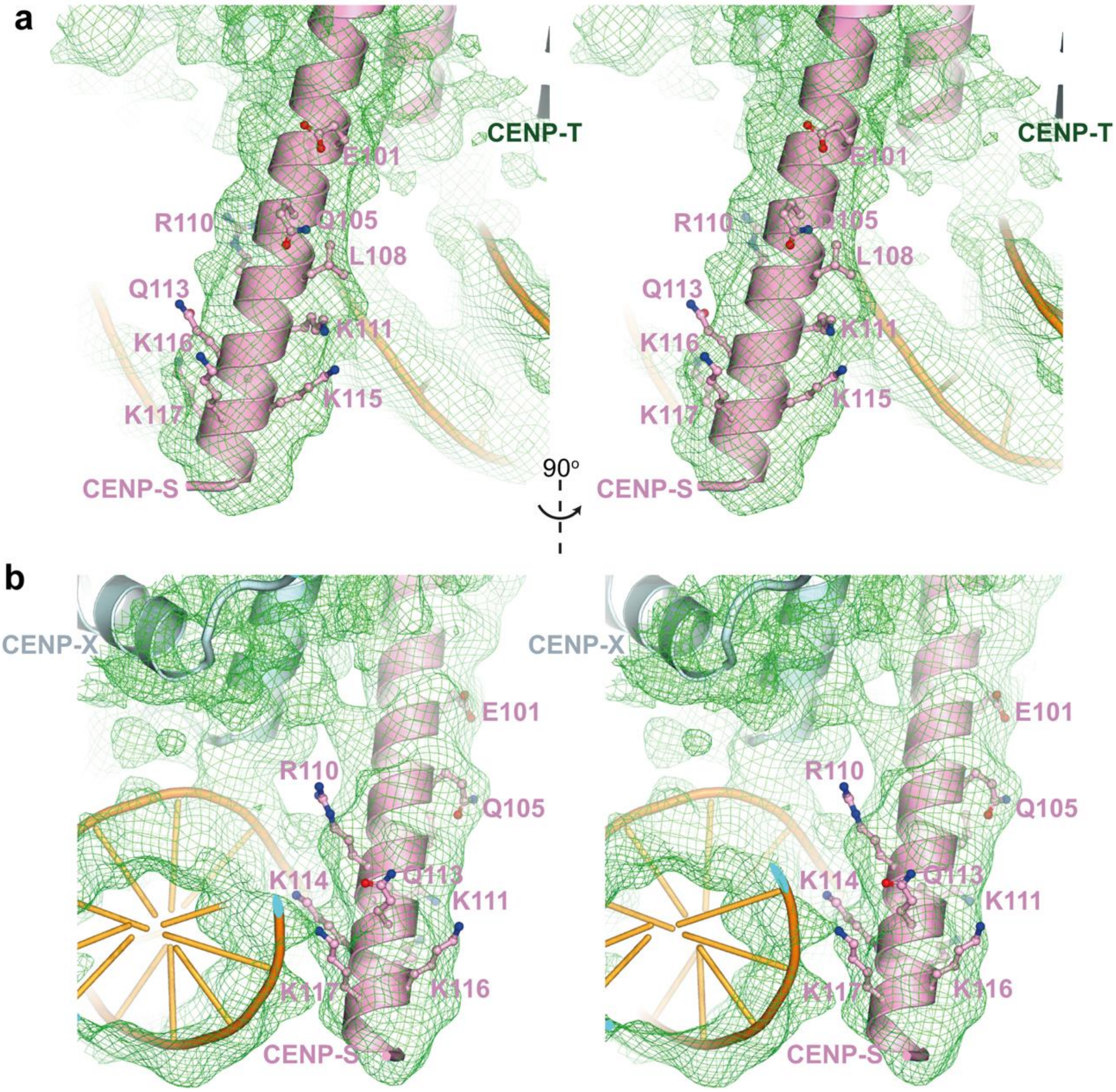

Supplementary Figure 3. Cryo-EM density fits for CENP-S α-helix of the CCAN:DNA complex. a, and **b**, Orthogonal stereo views of cryo-EM density map for the ordered C-terminal α-helix of CENP-S. Residues shown in Fig. 2c are indicated. View in (**a**) as in Fig. 2c.

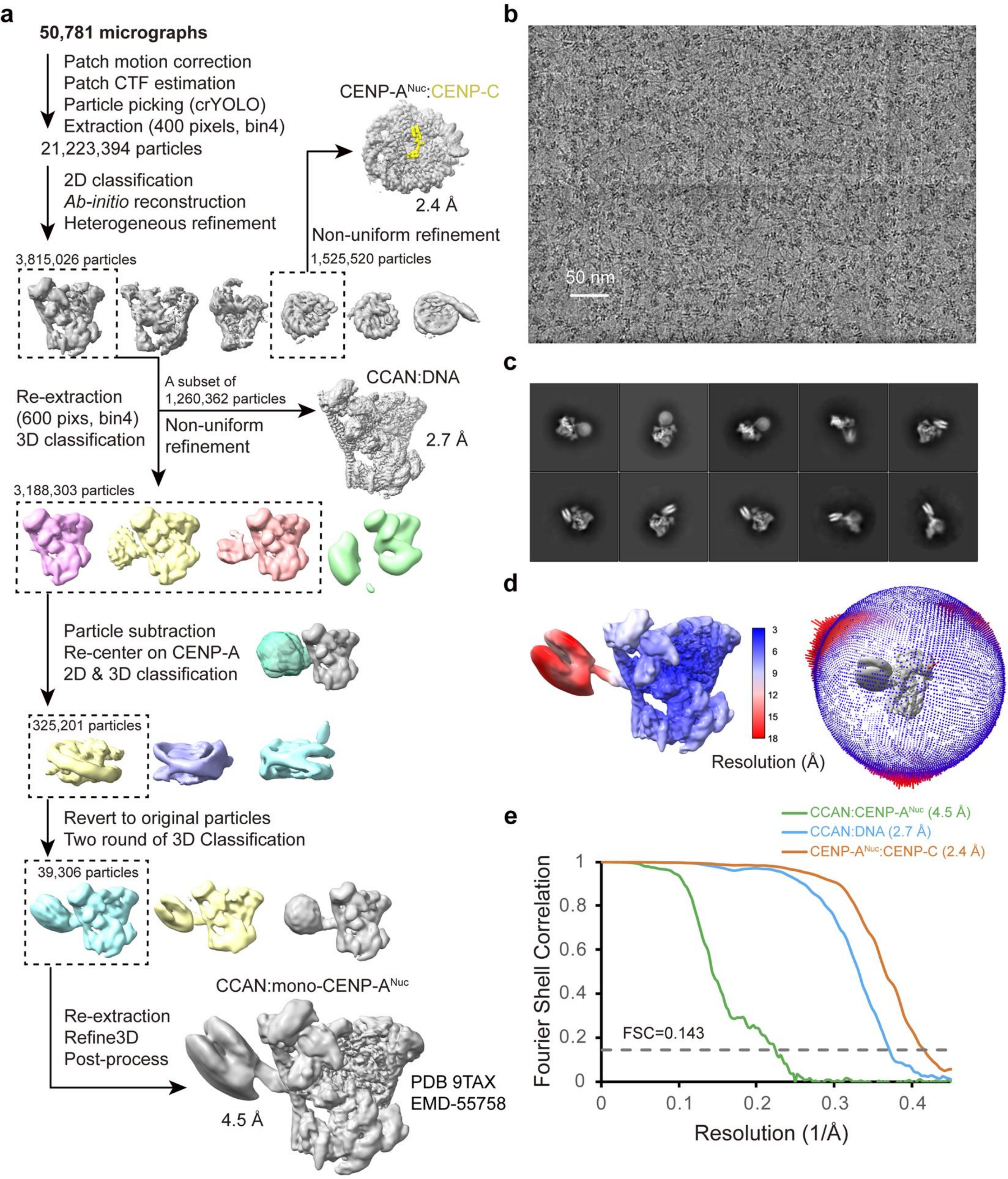

Supplementary Figure 4. Cryo-EM images and processing workflow for CCAN:mono-CENP-A^{Nuc} complex. **a**, Cryo-EM processing workflow. **b**, Representative cryo-electron micrograph of 50,781 collected. **c**, 2D class average gallery. **d**, Cryo-EM reconstruction colour-coded according to local resolution and plot of the angular distribution of particles used in the final reconstruction. **e**, FSC curves. The CCAN:DNA coordinates and map were deposited with codes PDB 28OP and EMD-56683, respectively. The CENP-A^{Nuc}:CENP-C coordinates and map were not deposited at PDB/RCSB with this study.

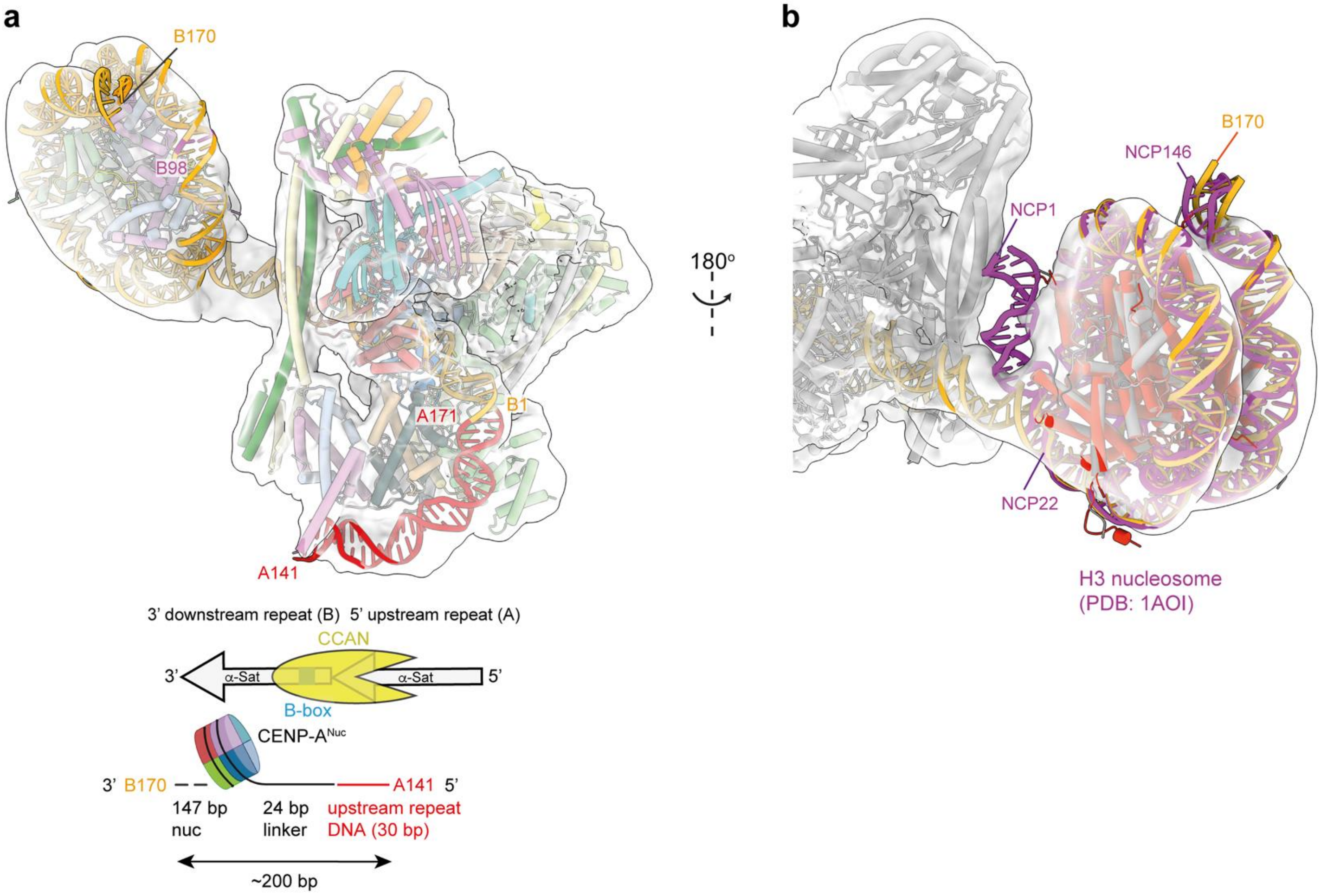

Supplementary Figure 5. Cryo-EM density fits for DNA of the CCAN:CENP-A^{Nuc} complex. a, Cryo-EM map of CCAN:CENP-A^{Nuc} complex with CCAN density in grey and DNA density in red (5' upstream repeat (A)) and orange (3' downstream repeat (B)). A141 and B170 refer to the positions on a dimeric α-satellite repeat sequence shown in the schematic below and as defined in Methods. B98 indicates the dyad axis of CENP-ANuc. **b**, Superimposition of a canonical H3 nucleosome (NCP) (DNA in magenta, histones in red) onto the CENP-A nucleosome (DNA in orange, histones in grey). This illustrates that ~20 bp of CENP-A^{Nuc} is unwrapped as the DNA gyre enters the CCAN DNA-binding tunnel. Nucleosome core particle (NCP) schematic in (a) Created in BioRender. Barford, D. (2026) https://BioRender.com/w602rxe.

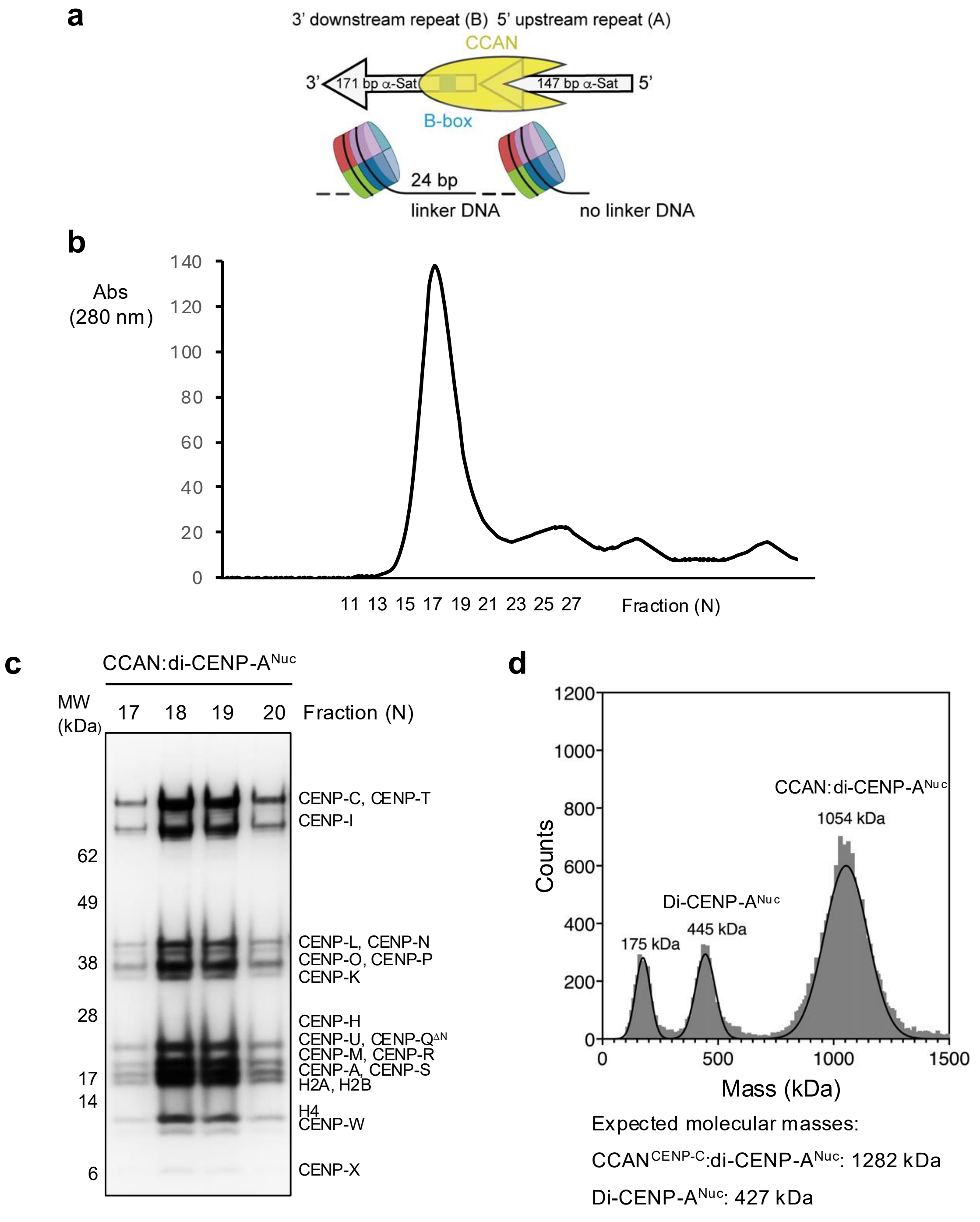

Supplementary Figure 6. Reconstitution of CCAN:di-CENP-A^{Nuc} complex. a, Schematic of CCAN:di-CENP-A^{Nuc} complex showing α-satellite repeat dimer used in this study. The 3' (downstream) repeat is 171 bp whereas the 5' upstream repeat is 147 bp, i.e. without linker DNA. This design ensures only one CCAN-binding linker DNA per α-satellite repeat dimer. **b** and **c**, Size exclusion chromatogram (b) and associated SDS PAGE gel (c) of purified CCAN:di-CENP-A^{Nuc} complex. **d**, iSCAT data for CCAN:di-CENP-A^{Nuc} complex showing a mixture of di-CENP-A^{Nuc} alone (445 kDa) and the CCAN:di-CENP-A^{Nuc} complex (1,054 kDa). Source data are provided as a Source Data file. SDS PAGE gel in (c) run at least three times. ISCAT experiment in (d) performed three times. NCP schematic in (a) Created in BioRender. Barford, D. (2026) https://BioRender.com/w602rxe.

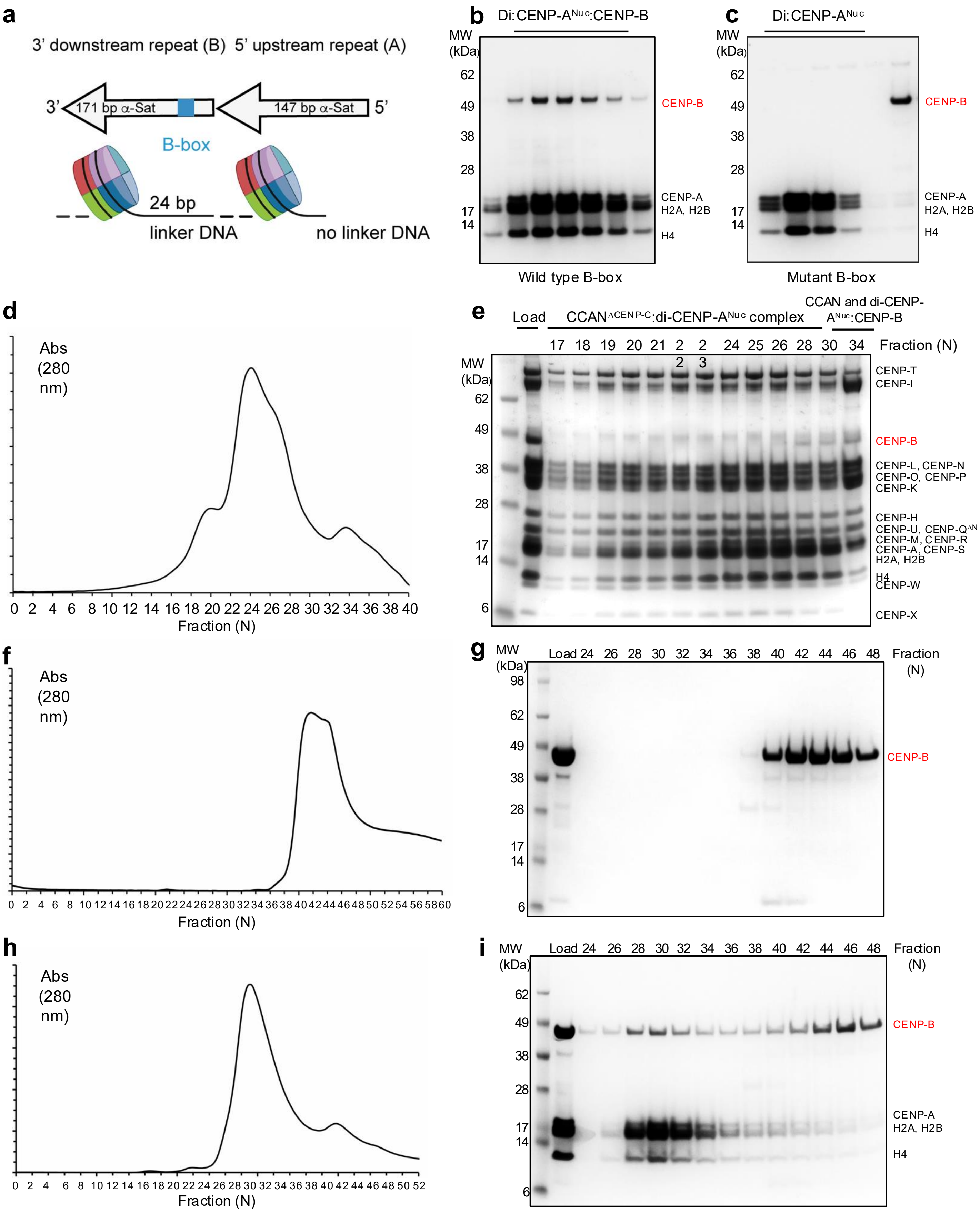

Supplementary Figure 7. CCAN association with linker DNA displaces CENP-B bound to linker DNA B-box. a, Schematic of the CCAN:di-CENP-A^{Nuc} complex showing α-satellite repeat dimer used in this study with B-box in blue. SDS PAGE gels of: **b**, di-CENP-A^{Nuc}:CENP-B complex (with wild type B-box) and **c**, showing CENP-B does not bind to di-CENP-A^{Nuc} with a mutated B-box. **d**, and **e**, Size exclusion chromatogram (d) and associated SDS PAGE gel (e) showing that CCAN binding to di-CENP-A^{Nuc} displaces CENP-B in a load sample of di-CENP-A^{Nuc}, CCAN and CENP-B (run on Agilent SEC-5 1000A 4.6x300 column). **f**, and **g**, Control SEC (f) and associated SDS PAGE gel (g) of CENP-B protein run on the same Agilent column. **h**, and **i**, Size exclusion chromatogram (h) and associated SDS PAGE gel (i) of di-CENP-A^{Nuc}:CENP-B complex on the same Agilent column. CENP-B in lanes 28-34 in panel (e) is from di-CENP-A^{Nuc}:CENP-B complex (compare with panel (i)). Source data are provided as a Source Data file. SDS PAGE gels in (b,c,e,g,i) run at least three times. NCP schematic in (a) Created in BioRender. Barford, D. (2026) https://BioRender.com/w602rxe.

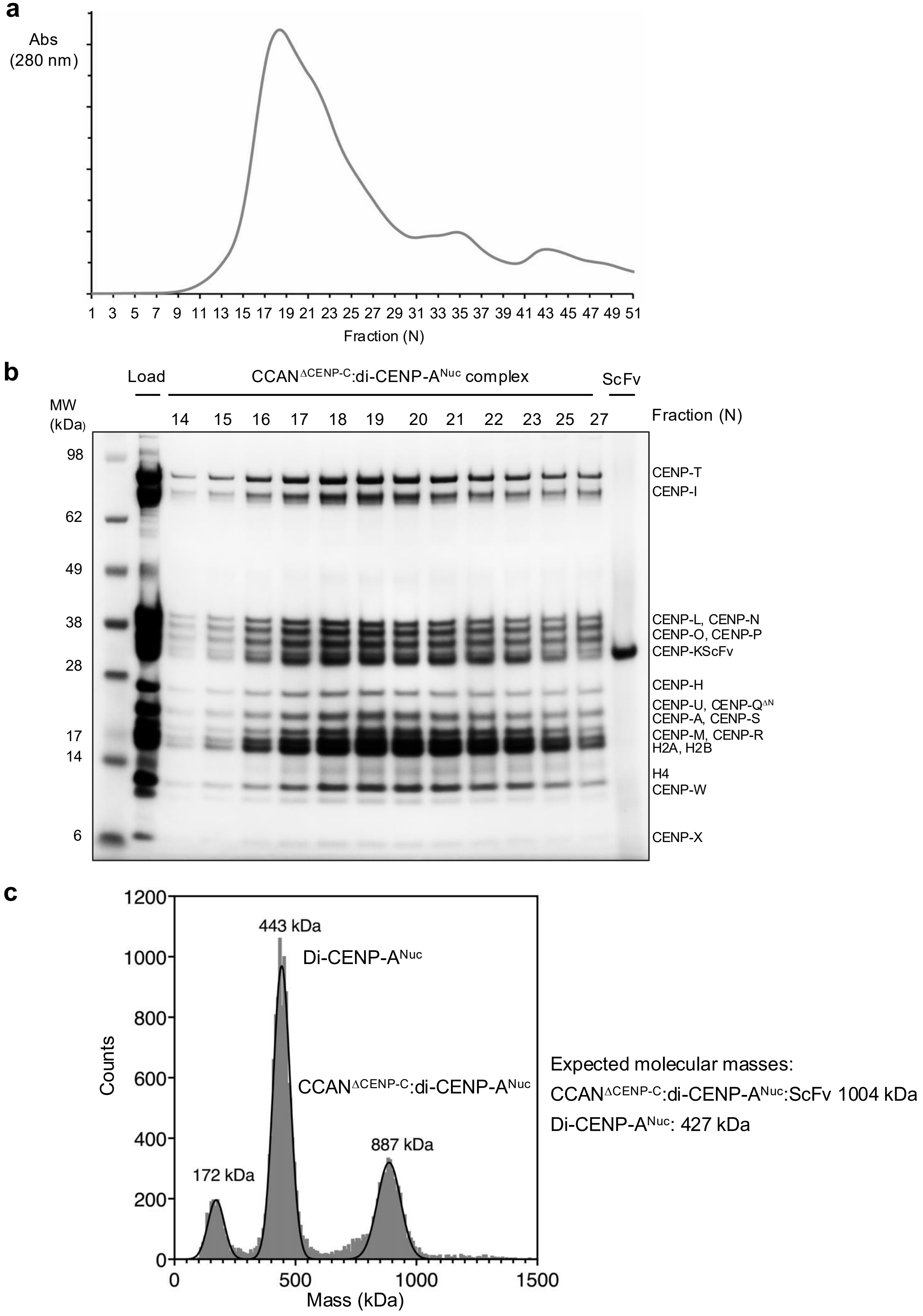

Supplementary Figure 8. Reconstitution of CCAN$^{DCENP\text{-}C}$:di-CENP-A^{Nuc} complex for cryo-EM. a and **b**, Size exclusion chromatogram (a) and associated SDS PAGE gel (b) of purified CCAN$^{\Delta CENP\text{-}C}$:di-CENP-A^{Nuc}:ScFv complex. **c**, iSCAT data for CCAN$^{\Delta CENP\text{-}C}$:di-CENP-A^{Nuc}:ScFv complex. Source data are provided as a Source Data file. SDS PAGE gel in (b) run at least three times. ISCAT experiment in (c) performed three times

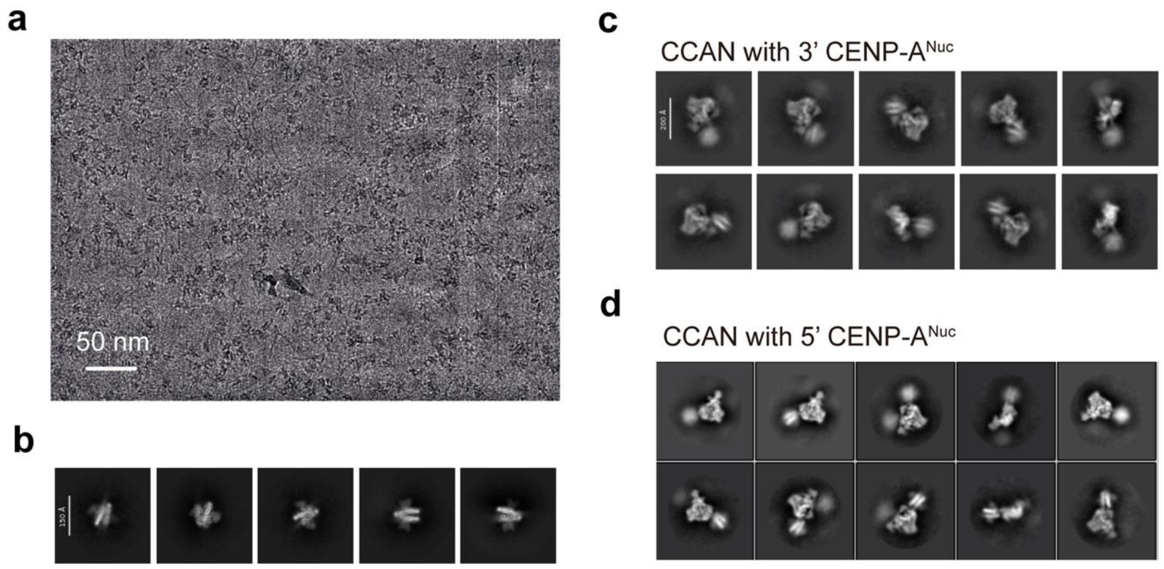

Supplementary Figure 9. Cryo-EM data for the CCAN:di-CENP-A^{Nuc} complex.
a, Representative cryo-electron micrograph of 28,703 collected. **b**, 2D class averages of CENP-A^{Nuc}. **c**, 2D class averages of CCAN with 3' CENP-A^{Nuc}. **d**, 2D class averages of CCAN with 5' CENP-A^{Nuc}.

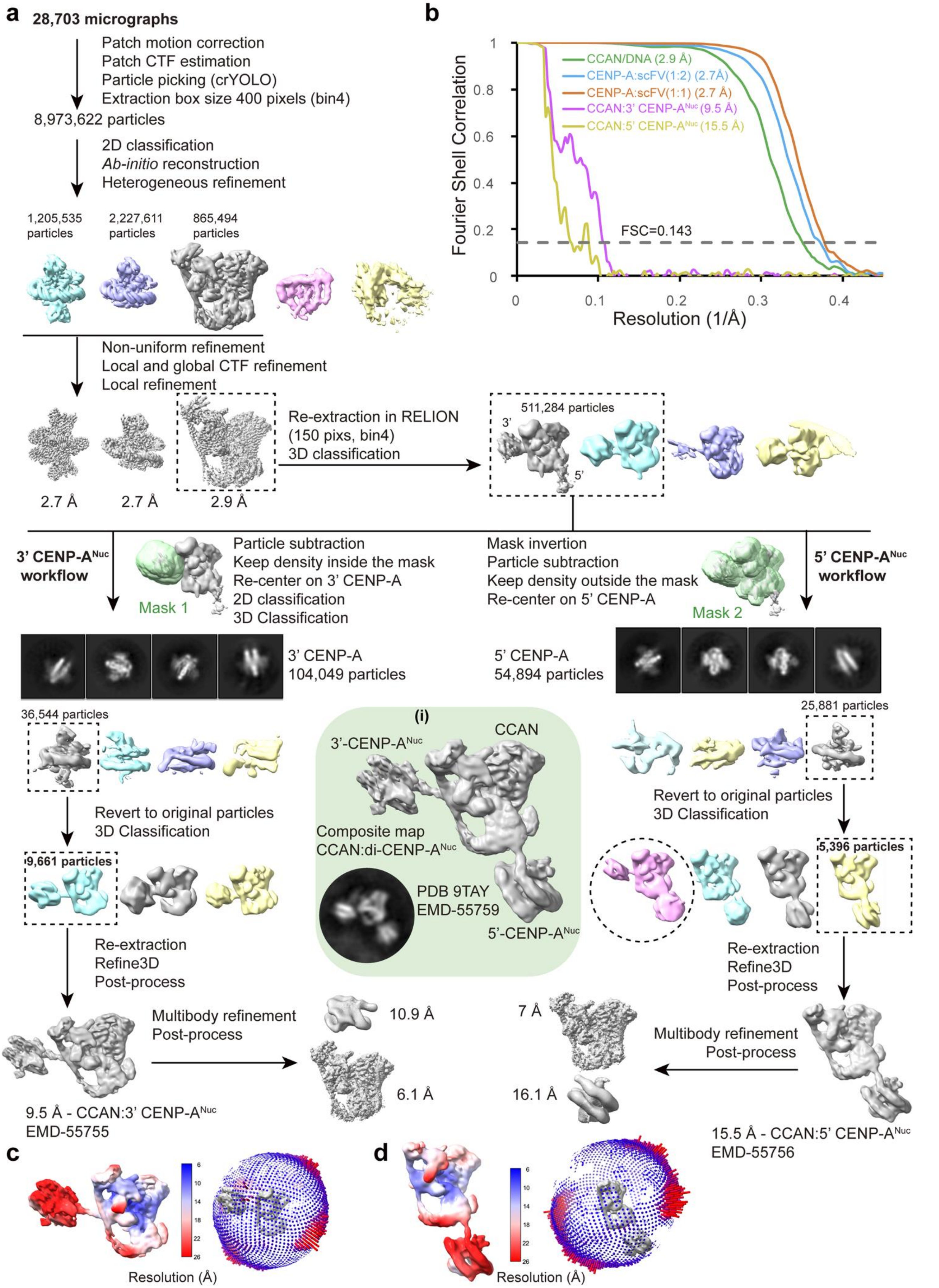

Supplementary Figure 10. Cryo-EM workflow for CCAN:di-CENP-A^{Nuc} complex reconstruction. a, Cryo-EM processing workflow with insert **(i)** showing the composite map and experimental 2D class average. **b**, FSC curves. Cryo-EM maps and coordinates for the CCAN:DNA, CENP-A^{Nuc}:ScFv (1:2) and CENP-A^{Nuc}:ScFv (1:1) complexes were not deposited with this study. **c**, Cryo-EM reconstruction colour-coded according to local resolution and plot of the angular distribution of particles used in the final reconstruction of CCAN:3'CENP-A^{Nuc}. **d**, Cryo-EM reconstruction colour-coded according to local resolution and plot of the angular distribution of particles used in the final reconstruction of CCAN:5'CENP-A^{Nuc}.

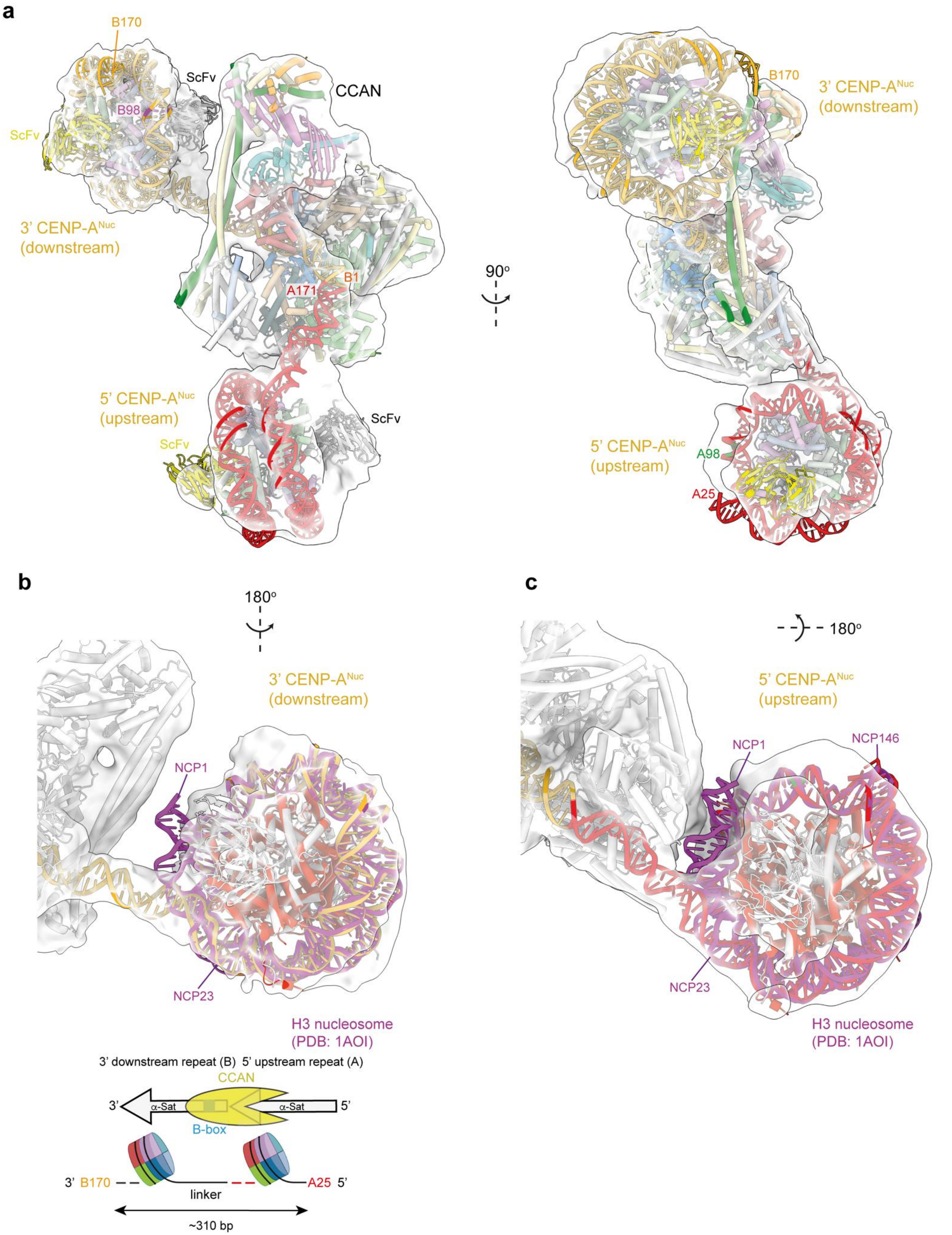

Supplementary Figure 11. Cryo-EM density fits for DNA of the CCAN:di-CENP-A^{Nuc} complex. a, Cryo-EM map of CCAN:di-CENP-A^{Nuc} complex with CCAN density in grey and DNA density in red (5’ upstream repeat (A)) and orange (3’ downstream repeat (B)). A25 and B170 refer to the positions on a dimeric α-satellite repeat sequence shown in the schematic below and as defined in Methods. B98 indicates the dyad axis of CENP-ANuc. **b**, Superimposition of a canonical H3 nucleosome (NCP) (DNA in magenta, histones in red) onto the CENP-A nucleosome (DNA in orange, histones in grey). This illustrates that ~20 bp of CENP-A^{Nuc} is unwrapped as the DNA gyre enters the CCAN DNA-binding tunnel. NCP schematic in (b) Created in BioRender. Barford, D. (2026) https://BioRender.com/w602rxe.

Supplementary Table 1. Cryo-EM data collection, refinement, and validation statistics

Complex	CCAN:DNA<br>PDB 9TAW<br>EMD-55757 (Supplementary 1c and sharpened map Fig. 1c)	CCAN:mono-CENP-A^{Nuc}<br>PDB 9TAX<br>EMD-55758	CCAN:di-CENP-A^{Nuc}<br>PDB 9TAY<br>EMD-55755, EMD55756, EMD-55759 (composite map), EMD-56612 (consensus map) (Supplementary Fig. 10)	
**Data collection and Processing**				
Microscope	Titan Krios	Titan Krios	Titan Krios	Titan Krios
Voltage (keV)	300	300	300	300
Camera	K3	K3	K3	K3
Magnification	105,000	81,000	81,000	81,000
Pixel size at detector (Å/pixel)	0.725	1.072	0.928	0.928
Total electron exposure (e^{-}/Å^2)	40	40	40	40
Exposure rate (e-/pixel/sec)	25	15	18	18
Number of frames	40	40	82	82
Defocus range (µm)	0.8-2.2	1-2	1.0-2.0	1.0-2.0
Automation software	EPU	EPU	EPU	EPU
Energy filter slit width (eV)	20	20	20	20
Micrographs collected (no.)	10,628	50,781	28,703	28,703
Total extracted particles (no.)	1,963,587	21,223,394	8,973,622	8,973,622
**For each reconstruction:**			CCAN:3'-CENP-A^{Nuc}	CCAN:5'-CENP-A^{Nuc}
Final particles (no.)	80,236	39,306	9,661	5,396
Point-group	C1	C1	C1	C1
Resolution (global, Å)	3.54	4.5	9.5	15.5
Resolution range (local, Å)	3.54-3.20	4.5-4.18	9.5-9.20	15.5-15.20
Map sharpening *B* factor (Å^2)	-73	-20	-50	-50
Map sharpening methods	cryoSPARC	RELION5	RELION5	RELION5
**Model composition**				
Protein (residues)	3165	3962	5565	
RNA/DNA (nucleotides)	162	398	636	
**Refinement**				
Refinement package	PHENIX 1.20.1	PHENIX 1.20.1	PHENIX 1.21	
real or reciprocal space	real	real	real	
Initial model used (Alphafold2)	7R5S/AlphaFold3	7YWX	7R5S/6E0P	
Model-Map scores				
CC Volume/Mask	0.74/0.77	0.80/0.80	0.46/0.55	
B factors (Å^2)				
Protein residues	113.26	358.74	488.77	
RNA/DNA	278.79	854.25	832.12	
R.m.s. deviations from ideal values				
Bond lengths (Å)	0.004	0.004	0.008	
Bond angles (°)	0.740	0.652	0.674	
**Validation**				
MolProbity score	1.52	1.73	1.82	
CaBLAM outliers	1.22	1.19	1.87	
Clashscore	6.70	14.00	13.12	
Poor rotamers (%)	0	0	0	
C-beta deviations	0	0	0	
EMRinger score	1.26	0.47	*n.d.	
Ramachandran plot				
Favored (%)	97.16	97.63	96.85	
Allowed (%)	2.78	2.3	3.12	
Outliers (%)	0.06	0.08	0.04	

Supplementary Table 2. DNA oligonucleotide primers used in this study

**Primer name**	**Sequence**
Primers for modification of pU1 for CENP-Q^{55-268} and CENP-$U^{235-418}$ expression	
QUF1	ATATGACUAA GCACACCAAC CTGAAG
QUR2	AGCGAGGUAT GTAGGCGGTG
QUF2	ACCTCGCUCT GCTAATCCTG
QUR	ATCATGGUCC ACATCTGGTG CCCCGA
QUF	ACCATGAUTG TAAATAAAAT GTAATTTACA GTA
QUR1	AGTCATAUTT ATAGGTTTTT TTATTACAAA ACTG
Primers for modification of pU1 for CENP-I isoform 1 expression	
cenpIF	ACTGTCCUTT CTTCTGGGCT TTCCTGTAGC AAAAAGGAAA CAGCGG
cenpIR	AGGACAGUAA ATGTGAGCTC TATTCGGGGA AAGAAATGGA GCTGGTATTT
Primers for cloning CENP-B into pET28 plasmid	
ricf	ATTCTGCUAA CCAGTAAGGC AAC
tevR	AGACTGGAAG UACAGGTTTT CTC
cbf	ACTTCCAGTC UATGGATAGT CTTGAATTCA TTGCTAGCAA GCTCGCAATG GGTCCGAAAC GTCGCCA
cbr	AAAGCTTUAA TGATGGTGAT GGTGATGGCT CTGATGACCC AGACCAC
pETF	AAAGCTTUCT GACCATTTAA ACAC
ricr	AGCAGAAUGA ATCACCGATA CG
Primers for assembly of ASW14	
9R	AGCTGTCuAC GACCAATTGT CTAGTTTTTA TGTGAAGATA TTTCCTGATA TCATGCGGCC TTGACG
9F1	CTAG CACCGCTTAA ACGCACGTAC GCGCTGTCCC CCGCGTTTTA ACCGCCAAGGGGATTACTCC CTAGTCTCCT TGTAGTATCT GCAAGTGGAC ATTCGGAGCA C TTTGTGGC
9R1	GTGCTCCGAA TGTCCACTTG CAGATACTAC AAGGAGACTA GGGAGTAATC CCCTTGGCGG TTAAAACGCG GGGGACAGCG CGTACGTGCG TTTAAGCGGT GCTAG
9F2	CTTCGTTGGAA ACGGGAATAT GTTCACATAA AAACTAGACA ATTGGTCGTA GACAGCTCTAGCACCGCTTA AACGCACGTA CGCGCTGTCC CCCGCGTTTT AACCGCCAAG G GGATTACT
9R2	CCTTGGCGGT TAAAACGCGG GGGACAGCGC GTACGTGCGT TTAAGCGGTG CTAGAGCTGT CTACGACCAA TTGTCTAGTT TTTATGTGAA CATATTCCCG TTTCCAACGA AGGCCACAAA
9F	GGATTACu CCCTAGTCTCC TTGTAGTATC TGGAATTGGA CATTTGGAGA TATCCTGGGC CTCATGGG
Primers for cloning ASW6	
ASW6R	AAAGAGTGAu ATCATGCGGC CTTGACG
ASW9F	*atc*ACTCTTu TTGTAGTATCTGCAAGTGGA CAT
