# Supplementary material for: Models for the architecture of the human inner kinetochore on centromeric α-satellite CENP-A nucleosome arrays
Source: Nat Commun. 2026 May 12;17:6346. doi: 10.1038/s41467-026-72856-0 (PMC13376190; doi:10.1038/s41467-026-72856-0)
Supplement: Supplementary file 2 — Description of Additional Supplementary Files [file 41467_2026_72856_MOESM2_ESM.pdf]

Description of Additional Supplementary Files

File Name: Supplementary Movie 1

Description: Video illustrates the difference in position of the 3' CENP-A nucleosome between our previous CCAN:CENP-A^{Nuc} complex with 171 bp DNA (grey cartoon) 12 and the structure of CCAN:CENP-A^{Nuc} with 211 bp DNA (ASW6) (colour-coded according to subunits as in Fig. 1). Relative to the previous CCAN:CENP-A^{Nuc} structure, the DNA register of the α-satellite repeat is shifted 6 bp (translation of 20 Å) away from CCAN (towards the 3' end). Because the DNA phosphate backbone contacts with CCAN are preserved, this results in a 216 deg rotation of CENP-A^{Nuc}.
