# Supplementary material for: Models for the architecture of the human inner kinetochore on centromeric α-satellite CENP-A nucleosome arrays
Source: Nat Commun. 2026 May 12;17:6346. doi: 10.1038/s41467-026-72856-0 (PMC13376190; doi:10.1038/s41467-026-72856-0)
Supplement: Supplementary file 4 — Reporting Summary [file 41467_2026_72856_MOESM4_ESM.pdf]

nature portfolio

Corresponding author(s): David Barford

Last updated by author(s): 17th Apri, 2026

Reporting Summary

Nature Portfolio wishes to improve the reproducibility of the work that we publish. This form provides structure for consistency and transparency in reporting. For further information on Nature Portfolio policies, see our Editorial Policies and the Editorial Policy Checklist.

Statistics

For all statistical analyses, confirm that the following items are present in the figure legend, table legend, main text, or Methods section.

n/a	Confirmed	
☐	☒	The exact sample size (*n*) for each experimental group/condition, given as a discrete number and unit of measurement
☐	☒	A statement on whether measurements were taken from distinct samples or whether the same sample was measured repeatedly
☒	☐	The statistical test(s) used AND whether they are one- or two-sided<br>*Only common tests should be described solely by name; describe more complex techniques in the Methods section.*
☒	☐	A description of all covariates tested
☒	☐	A description of any assumptions or corrections, such as tests of normality and adjustment for multiple comparisons
☒	☐	A full description of the statistical parameters including central tendency (e.g. means) or other basic estimates (e.g. regression coefficient) AND variation (e.g. standard deviation) or associated estimates of uncertainty (e.g. confidence intervals)
☒	☐	For null hypothesis testing, the test statistic (e.g. *F*, *t*, *r*) with confidence intervals, effect sizes, degrees of freedom and *P* value noted<br>*Give P values as exact values whenever suitable.*
☒	☐	For Bayesian analysis, information on the choice of priors and Markov chain Monte Carlo settings
☒	☐	For hierarchical and complex designs, identification of the appropriate level for tests and full reporting of outcomes
☒	☐	Estimates of effect sizes (e.g. Cohen's *d*, Pearson's *r*), indicating how they were calculated

Our web collection on statistics for biologists contains articles on many of the points above.

Software and code

Policy information about availability of computer code

Data collection	EM data collection Commercial software was used: EPU (version 3.4.0.5704 REL) from Thermo Fisher Scientific for automated cryo-EM data collection.
Data analysis	Micrograph movie frames were aligned with MotionCor2. CTF estimation was performed using CTFFIND4 v4.1.14 . Data were processed using RELION v5.0 and cryoSPARC v4.7.1. Particle picking was performed with crYOLO v1.9.9. Models were built using AlphaFold (https://alphafold.ebi.ac.uk/), Chimera X v1.10 and COOT v0.8.9.2 and real-space refinement in PHENIX v1.20.1. Model validation was performed using MolProbity v4.5. Visualization was done using Chimera X v1.4. Sequence alignments were performed using Uniprot, Clutal Omega (https://www.ebi.ac.uk/Tools/msa/clustalo/) and displayed with JALVIEW v 1.0. Structure prediction was performed using local installation of the ALPHAFOLD3 software. ISCAT software (REFYN) software:AcquireMP 2025 R1, DiscoverMP v2025 R1, PRISM 10.6.1.

For manuscripts utilizing custom algorithms or software that are central to the research but not yet described in published literature, software must be made available to editors and reviewers. We strongly encourage code deposition in a community repository (e.g. GitHub). See the Nature Portfolio guidelines for submitting code & software for further information.

Data

Policy information about availability of data

All manuscripts must include a data availability statement. This statement should provide the following information, where applicable:

- Accession codes, unique identifiers, or web links for publicly available datasets
- A description of any restrictions on data availability
- For clinical datasets or third party data, please ensure that the statement adheres to our policy

CryoEM maps have been deposited with the Electron Microscopy Data Bank (EMDB) with accession codes: EMB-55755, EMD-55756, EMD-55757, EMB-55758, EMD-55759, EMD-56683. Protein coordinate have been deposited with RCSB with accession codes: 9TAW, 9TAX, 9TAY, 28OP.
PDB coordinates generated in this study have been deposited with RCSB under accession codes
9TAW [https://www.rcsb.org/structure/9TAW]
9TAX [https://www.rcsb.org/structure/9TAX]
9TAY [https://www.rcsb.org/structure/9TAY]
28OP [https://www.rcsb.org/structure/28OP].
Cryo-EM maps generated in this study have been deposited with EMDB under accession codes
EMD-55755 [https://www.ebi.ac.uk/emdb/EMD-55755]
EMD-55756 [https://www.ebi.ac.uk/emdb/EMD-55756]
EMD-55757 [https://www.ebi.ac.uk/emdb/EMD-55757]
EMD-55758 [https://www.ebi.ac.uk/emdb/EMD-55758]
EMD-55759 [https://www.ebi.ac.uk/emdb/EMD-55759]
EMD-56683 [https://www.ebi.ac.uk/emdb/EMD-56683]
Previously published protein coordinates used in study:
7R5S [https://www.rcsb.org/structure/7R5S],
7YWX [https://www.rcsb.org/structure/7YWX]
6E0P [https://www.rcsb.org/structure/6E0P]
1AOI [https://www.rcsb.org/structure/1AOI].
Accession numbers are also listed in Supplementary Table 1. Source data are provided with this paper. Correspondence and requests for materials should be addressed to David Barford.

Research involving human participants, their data, or biological material

Policy information about studies with human participants or human data. See also policy information about sex, gender (identity/presentation), and sexual orientation and race, ethnicity and racism.

Reporting on sex and gender	NA
Reporting on race, ethnicity, or other socially relevant groupings	NA
Population characteristics	NA
Recruitment	NA
Ethics oversight	NA

Note that full information on the approval of the study protocol must also be provided in the manuscript.

Field-specific reporting

Please select the one below that is the best fit for your research. If you are not sure, read the appropriate sections before making your selection.

☒ Life sciences ☐ Behavioural & social sciences ☐ Ecological, evolutionary & environmental sciences

For a reference copy of the document with all sections, see nature.com/documents/nr-reporting-summary-flat.pdf

Life sciences study design

All studies must disclose on these points even when the disclosure is negative.

Sample size	No statistical method was used for sample size calculation.<br>For the CCAN-DNA complex, a total of 10,628 micrographs were collected. A total number of 1,963,587 particles were auto-picked, of which 80,236 were used for a final reconstruction.<br>For the CCAN-mono-CENP-A nucleosome complex, a total of 50,781 micrographs were collected. A total number of 21,223,394 particles were auto-picked, of which 39,306 were used for a final reconstruction. For the CCAN-di-CENP-A nucleosome complex, a total of 28,703 micrographs were collected. of which 9,661 were used for CCAN:3’ CENP-A reconstruction and 5,396 were used for CCAN:5’ CENP-A reconstruction.

For cryoEM reconstructions, sample sizes were determined by available electron microscopy time and the number of particles on each micrograph obtained during the collection time.

Data exclusions	Through 2D and 3D classification procedures, broken particles or particles that do not belong to the classes of interests were discarded. This is a standard practice in cryoEM studies to obtain homogeneous cryoEM structures.
Replication	CryoEM datasets were collected with multiple samples in separate imaging sessions. All biochemical experiments were repeated at least in three independent experiments and are all reproducible.
Randomization	No randomization was performed, since this study did not allocate experimental groups.
Blinding	No blinding was performed, since it is not relevant to this study.

Reporting for specific materials, systems and methods

We require information from authors about some types of materials, experimental systems and methods used in many studies. Here, indicate whether each material, system or method listed is relevant to your study. If you are not sure if a list item applies to your research, read the appropriate section before selecting a response.

Materials & experimental systems

n/a	Involved in the study	
☒	☐	Antibodies
☐	☒	Eukaryotic cell lines
☒	☐	Palaeontology and archaeology
☒	☐	Animals and other organisms
☒	☐	Clinical data
☒	☐	Dual use research of concern
☒	☐	Plants

Methods

n/a	Involved in the study	
☒	☐	ChIP-seq
☒	☐	Flow cytometry
☒	☐	MRI-based neuroimaging

Eukaryotic cell lines

Policy information about cell lines and Sex and Gender in Research

Cell line source(s)	High-5 insect cell (BTI-TN-5B1-4) were obtained from Thermo Fisher Scientific (Cat. No. 10486-025).
Authentication	The High-5 insect cell line was not authenticated
Mycoplasma contamination	The High-5 insect cell line was not tested for mycoplasma contamination.
Commonly misidentified lines (See ICLAC register)	None
