# Supplementary material for: Models for the architecture of the human inner kinetochore on centromeric α-satellite CENP-A nucleosome arrays
Source: Nat Commun. 2026 May 12;17:6346. doi: 10.1038/s41467-026-72856-0 (PMC13376190; doi:10.1038/s41467-026-72856-0)
Supplement: Supplementary file 5 — Transparent Peer Review file [file 41467_2026_72856_MOESM5_ESM.pdf]

nature portfolio

Peer Review File

Models for the architecture of the human inner kinetochore on centromeric α-satellite CENP-A nucleosome arrays

Corresponding Author: Dr David Barford

This manuscript has been previously reviewed at another journal. This document only contains information relating to versions considered at Nature Communications. Mentions of the other journal have been redacted.

This file contains all reviewer reports in order by version, followed by all author rebuttals in order by version.

Version 1:

Reviewer comments:

Reviewer #1

(Remarks to the Author)
In my opinion, the authors have addressed the concerns satisfactorily. Hence, I support the publication of this manuscript in Nature Communications.

Reviewer #2

(Remarks to the Author)
The authors appropriately responded to our previous criticisms. This is an important contribution to the centromere biology, and so we would like to support publication of this manuscript after addressing some minor points listed below.

1. Fig. 1a and Supplementary Fig. 2
The authors assigned specific DNA segment (A140-B40) to the resolved cryo-EM density, but it is impossible to validate this assignment based on the presented maps. It would be helpful to include additional close-up maps to justify the base assignment. This will also justify the statements in Lines 216-220, relating to the map quality.

2. In lines 113-116, the authors stated, " However, in the new structure, we observed an additional 20 bp of DNA exiting the DNA binding tunnel, so that in total 40 bp of DNA interacts with the entire CENP-TWSX module. Additionally, a short segment of DNA (~5 bp) extends beyond the CENP-TWSX module and approaches CENP-QU.". We assume that the additional 20 bp refers to the extension from A161 to A140. It is not clear where did "~5 bp" come from.

3. Supplementary Fig.2a. EMDB-14331 must be a typo, as it refers to an unrelated structure " Core-binding domain of fungal E3-binding domain bound to the pyruvate dehydrogenase E2 core".

4. Supplementary Fig.2a and 2b.
The white rectangular annotation of CENP-S C-term alpha-helix is confusing as it is the same color of the cryo-EM density map. Superimposing the transparent density to the model with a different color would suffice the purpose. Similar presentation can be done for overlaying the density to the DNA model, as noted above.

5. Supplementary Fig.3b. The character "b" is trimmed.

6. Lines 171-172. "Both individual CCAN:DNA and CENP-ANuc modules were resolved in cryo-EM density, showing relative flexibility (Supplementary Fig 3d)." This is a cryptic sentence. We assume that the authors intend to note that cryo-EM densities of CCAN:DNA and CENP-ANuc can be resolved individually, but they cannot achieve high resolutions simultaneously (also based on Supplementary Fig 3e), indicating that their connection is flexible. Please explain this clearer.

7. Fig. 1d
Abbreviations "-ve" and "+ive" are not immediately intuitive.

Reviewer #3

(Remarks to the Author)
I co-reviewed this manuscript with one of the reviewers who provided the listed reports. This is part of the Nature Communications initiative to facilitate training in peer review and to provide appropriate recognition for Early Career Researchers who co-review manuscripts.

Reviewer #4

(Remarks to the Author)
This manuscript provides further clarification of the di-CENP-A nucleosome-CCAN structure, building upon the CCAN structure that was previously elucidated by the authors' group through cryo-EM single-particle analysis. We also reviewed the authors' previous manuscript submitted to [*REDACTED*] (Reviewer 4).

The novel aspects of this paper are as follows:
1. It clearly demonstrates the binding mode of CENP-TWSX to DNA.
2. It reveals a structure that suggests a binding preference when either CCAN or the CENP-A nucleosome-CCAN complex binds to alpha-satellite repeat sequences.

While the di-CENP-A nucleosome-CCAN structure is interesting, it is unclear what cellular conditions it reflects. However, it is currently difficult to demonstrate this experimentally.

I believe the authors have addressed the reviewers' comments on the previous manuscript.

Although the manuscript has been lengthened by the reviewers' suggestions, the revised discussion (lines 485-554) seems excessive in relation to the presented data and may be difficult for general readers to understand. All three models are described as potentially consistent with the previously reported copy numbers of CENP-A, CENP-C, and CENP-T proteins per centromere (Suzuki et al., lines 497–503). However, the subsequent discussion (lines 502-508) makes it difficult to evaluate this consistency. This section (lines 485–508) could be shortened by focusing more on the strengths and weaknesses of the models. Additionally, the description in lines 485–487 is repeated in lines 507–509.

Regarding comment 2 from Reviewer 4, the stoichiometry among the CENP-C dimer, the CENP-A nucleosome, and the CCAN is unclear, as the authors themselves note (lines 490–497). It may be difficult to discuss the stoichiometry of CENP-C in the proposed models, as well as its role in the higher-order organization of the kinetochore, solely based on the structural data presented here (lines 510-523). These issues could be discussed alongside the preceding section (lines 490–497) as a future perspective, for example.

Open Access This Peer Review File is licensed under a Creative Commons Attribution 4.0 International License, which permits use, sharing, adaptation, distribution and reproduction in any medium or format, as long as you give appropriate credit to the original author(s) and the source, provide a link to the Creative Commons license, and indicate if changes were made.
In cases where reviewers are anonymous, credit should be given to 'Anonymous Referee' and the source.
The images or other third party material in this Peer Review File are included in the article's Creative Commons license, unless indicated otherwise in a credit line to the material. If material is not included in the article's Creative Commons license and your intended use is not permitted by statutory regulation or exceeds the permitted use, you will need to obtain permission directly from the copyright holder.
To view a copy of this license, visit https://creativecommons.org/licenses/by/4.0/

NCOMMS-26-002471A. C. Yu et al.

REVIEWERS' COMMENTS

Reviewer #1 (Remarks to the Author):

In my opinion, the authors have addressed the concerns satisfactorily. Hence, I support the publication of this manuscript in Nature Communications.

We thank the referee for reviewing the manuscript and for recommending publication.

Reviewer #2 (Remarks to the Author):

The authors appropriately responded to our previous criticisms. This is an important contribution to the centromere biology, and so we would like to support publication of this manuscript after addressing some minor points listed below.

We thank the referee for reviewing the manuscript and for recommending publication and for additional suggestions to improve the manuscript.

1. Fig. 1a and Supplementary Fig. 2
The authors assigned specific DNA segment (A140-B40) to the resolved cryo-EM density, but it is impossible to validate this assignment based on the presented maps. It would be helpful to include additional close-up maps to justify the base assignment. This will also justify the statements in Lines 216-220, relating to the map quality.

We have generated a new figure (Supplementary Fig. 2c) of close-up views of the maps showing cryo-EM density.

2. In lines 113-116, the authors stated, " However, in the new structure, we observed an additional 20 bp of DNA exiting the DNA binding tunnel, so that in total 40 bp of DNA interacts with the entire CENP-TWSX module. Additionally, a short segment of DNA (~5 bp) extends beyond the CENP-TWSX module and approaches CENP-QU.". We assume that the additional 20 bp refers to the extension from A161 to A140. It is not clear where did "~5 bp" come from.

This was a poorly phrased statement. Now revised. The "~5 bp" is included within the additional 20 bp. Lines: 120-121.

3. Supplementary Fig.2a. EMDB-14331 must be a typo, as it refers to an unrelated structure " Core-binding domain of fungal E3-binding domain bound to the pyruvate dehydrogenase E2 core".

Thank you for this. It should be EMDB-14336. Now revised.

4. Supplementary Fig.2a and 2b.
The white rectangular annotation of CENP-S C-term alpha-helix is confusing as it is the same color of the cryo-EM density map. Superimposing the transparent density to the model with a different color would suffice the purpose. Similar presentation can be done for overlaying the density to the DNA model, as noted above.

To distinguish cryo-EM density of CCAN from the coordinates, we have coloured the CCAN coordinates blue in Supplementary Fig. 2a, b. Using ChimeraX it is not possible to colour transparent maps with the colour of the coordinates. We want to show the cryo-EM density of DNA in orange as a separate colour from the CCAN cryo-EM density (grey). Our new colour scheme clearly shows that the CENP-S C-term a-helix is ordered in the CCAN:DNA complex with the longer DNA (Supplementary Fig. 2a), but not in the previous CCAN:DNA complex (Supplementary Fig. 2b).

5. Supplementary Fig.3b. The character "b" is trimmed.

Corrected

6. Lines 171-172. "Both individual CCAN:DNA and CENP-ANuc modules were resolved in cryo-EM density, showing relative flexibility (Supplementary Fig 3d)." This is a cryptic sentence. We assume that the authors intend to note that cryo-EM densities of CCAN:DNA and CENP-ANuc can be resolved individually, but they cannot achieve high resolutions simultaneously (also based on Supplementary Fig 3e), indicating that their connection is flexible. Please explain this clearer.

Thank for this. The sentence has been revised to:

Both individual CCAN:DNA and CENP-A^{Nuc} modules were resolved in the consensus map, with the CCAN:DNA module defined to a higher resolution than the $CENP^{Nuc}$ module, indicating a flexible connection between them (Supplementary Fig. 4d). lines 154-156.

7. Fig. 1d
Abbreviations "-ve" and "+ive" are not immediately intuitive.

Changed to "-" and "+".

Reviewer #3 (Remarks to the Author):

I co-reviewed this manuscript with one of the reviewers who provided the listed reports. This is part of the Nature Communications initiative to facilitate training in peer review and to provide appropriate recognition for Early Career Researchers who co-review manuscripts.

Reviewer #4 (Remarks to the Author):

This manuscript provides further clarification of the di-CENP-A nucleosome-CCAN structure, building upon the CCAN structure that was previously elucidated by the authors' group through cryo-EM single-particle analysis. We also reviewed the authors' previous manuscript submitted to [*REDACTED*]

(Reviewer 4).

The novel aspects of this paper are as follows:
1. It clearly demonstrates the binding mode of CENP-TWSX to DNA.
2. It reveals a structure that suggests a binding preference when either CCAN or the CENP-A nucleosome-CCAN complex binds to alpha-satellite repeat sequences.

While the di-CENP-A nucleosome-CCAN structure is interesting, it is unclear what cellular conditions it reflects. However, it is currently difficult to demonstrate this experimentally.

I believe the authors have addressed the reviewers' comments on the previous manuscript.

Although the manuscript has been lengthened by the reviewers' suggestions, the revised discussion (lines 485-554) seems excessive in relation to the presented data and may be difficult for general readers to understand. All three models are described as potentially consistent with the previously reported copy numbers of CENP-A, CENP-C, and CENP-T proteins per centromere (Suzuki et al., lines 497–503). However, the subsequent discussion (lines 502-508) makes it difficult to evaluate this consistency. This section (lines 485–508) could be shortened by focusing more on the strengths and weaknesses of the models. Additionally, the description in lines 485–487 is repeated in lines 507–509.

Regarding comment 2 from Reviewer 4, the stoichiometry among the CENP-C dimer, the CENP-A nucleosome, and the CCAN is unclear, as the authors themselves note (lines 490–497). It may be difficult to discuss the stoichiometry of CENP-C in the proposed models, as well as its role in the higher-order organization of the kinetochore, solely based on the structural data presented here (lines 510-523). These issues could be discussed alongside the preceding section (lines 490–497) as a future perspective, for example.

We thank the referee for reviewing the manuscript and for additional suggestions to improve the manuscript.

We have revised and shortened the Discussion and include a new schematic figure (Fig. 6) that indicates how the three models of the CCAN:CENP-A nucleosome assembly are arranged on alpha-satellite repeat sequences (lines 434-456).
